# The multifaceted perspectives on the regulation of lncRNAs in hepatocellular carcinoma ferroptosis: from bench-to-bedside

**DOI:** 10.1007/s10238-024-01418-9

**Published:** 2024-07-03

**Authors:** Xin Jin, Chun Xia Huang, Yue Tian

**Affiliations:** Department of Gastroenterology and Hepatology, Fengdu People’s Hospital, Fengdu County, Chongqing, 408200 China

**Keywords:** Hepatocellular carcinoma, LncRNA, Ferroptosis, Cell death, Cancer therapy

## Abstract

Despite being characterized by high malignancy, high morbidity, and low survival rates, the underlying mechanism of hepatocellular carcinoma (HCC) has not been fully elucidated. Ferroptosis, a non-apoptotic form of regulated cell death, possesses distinct morphological, biochemical, and genetic characteristics compared to other types of cell death. Dysregulated actions within the molecular network that regulates ferroptosis have been identified as significant contributors to the progression of HCC. Long non-coding RNAs (lncRNAs) have emerged as influential contributors to diverse cellular processes, regulating gene function and expression through multiple mechanistic pathways. An increasing body of evidence indicates that deregulated lncRNAs are implicated in regulating malignant events such as cell proliferation, growth, invasion, and metabolism by influencing ferroptosis in HCC. Therefore, elucidating the inherent role of ferroptosis and the modulatory functions of lncRNAs on ferroptosis in HCC might promote the development of novel therapeutic interventions for this disease. This review provides a succinct overview of the roles of ferroptosis and ferroptosis-related lncRNAs in HCC progression and treatment, aiming to drive the development of promising therapeutic targets and biomarkers for HCC patients.

## Introduction

Hepatocellular carcinoma (HCC), accounting for the vast majority (75–85%) of primary liver cancer, represents the sixth most prevalent cancer and ranks third in terms of cancer death worldwide [1, 2]. HCC is a devastating gastrointestinal malignancy characterized by invasive growth, high relapse tendency, and late diagnosis [3]. Its 5-year survival rate is only 18%, which poses a substantial burden on sustainable global healthcare [4]. The pathogenesis of HCC is attributed to hepatitis viral infections, metabolic syndrome, and genetic factors [1, 3]. Although the incidence rates of HCC have sharply declined over the past 30 years due to the significant improvement in the management of viral hepatitis, the emerging prevalence of non-viral factors such as diabetes and obesity potentially offset the efforts on HCC prevention [4, 5]. The prognosis of HCC patients depends on the disease stage to a large extent. Surgical resection or ablation are widely accepted treatment options with prolonged survival for early-stage patients [6]. For patients with advanced-stage HCC, systematic therapies inclusive of tyrosine kinase inhibitor (TKI) monotherapy or immunological combination therapy are preferred treatment modalities but yield unsatisfactory prognoses owing to limited therapeutic efficiency [6, 7]. This grim therapeutic response potentially stems from impaired cell death induction [8, 9]; thereby, a comprehensive understanding and evaluation of the underlying molecular mechanisms of cell death during HCC development might pave the way for promising therapies.

Cell death is an inevitable process of fundamental significance to ensure the normal development of the human body by eliminating nonfunctional and damaged cells [10]. It is not only a natural process that contributes to embryonal development and organismal homeostasis but also can be a pathological reaction in the context of damaging stimuli (e.g., physical injury and infection), which comes at the cost of chronic inflammation [10, 11]. Considering its morphological characteristics and molecular functions, cell death modes can be categorized into regulated cell death (RCD) and accident cell death (ACD) [11–13]. ACD is a nonregulated biological process elicited by unexpected injury stimuli, while RCD involves controlled signaling cascades and defined molecular effector mechanisms [10, 13]. Apoptosis was previously regarded as the major subtype of RCD, but accumulated experimental evidence has revealed several emerging forms of RCD, including necroptosis, pyroptosis, alkaliptosis, ferroptosis, and cuproptosis [14, 15]. Malignant cells have evolved myriad mechanisms to resist the RCD routes [16, 17]. In addition, RCD pathways play a central role in cancer biology inclusive of cancer metastasis and immune evasion [18, 19]. Based on emerging evidence, the RCD process releases massive danger-associated molecular patterns (DAMPs) or pathogen-associated molecular patterns (PAMPs), thereby changing the tumor microenvironment (TME) and affecting the benefits of anticancer treatment [20].

Ferroptosis, a non-apoptotic type of RCD, exhibiting distinct morphological, biochemical, and genetic characteristics compared to other types of cell death, is modulated by multiple cellular metabolic processes such as iron handling, glycolipid metabolism, and redox homeostasis, along with various signaling pathways associated with disease progression [21, 22]. Ferroptosis is characterized by the existence of diminished mitochondria and decreased mitochondrial cristae [23]. This mechanistic characteristic sets ferroptosis apart from other modes of RCD, which are governed by specific proteins that execute cell death [24]. Dysregulated actions in the molecular network that regulate ferroptosis have been recognized as significant contributors to the advancement of various diseases, including HCC [25–27]. It is important to highlight the pharmacological modulation of ferroptosis holding significant potential for HCC treatment [26, 27]. Moreover, some examples have revealed that ferroptosis plays an essential role in HCC therapy. For example, polyphyllin, a traditional Chinese medicine, could suppress HCC progression by inducing the ferroptosis pathway through the Nuclear respiration factor-2 (NRF2) /HO-1/ glutathione peroxidase 4 (GPX4) axis [28]. And brusatol could induce ferroptosis to suppresses HCC via targeting activating transcription factor 3 (ATF3) [29]. Therefore, comprehensively understanding the mechanistic actions that govern ferroptosis might pave the way for developing novel drugs for patients with HCC.

Long noncoding RNAs (lncRNAs) constitute a diverse category of non‐protein‐coding transcripts with lengths exceeding 200 nucleotides [30]. LncRNAs serve as emerging regulators involved in various cellular events including chromatin remodeling, transcriptional regulation, and epigenetic modification, which affect a wide range of physiological and pathological processes [31, 32]. Emerging studies have provided compelling evidence that lncRNAs play a crucial role in several aspects of biological activities [33, 34]. Dysregulated lncRNAs regulate ferroptosis-related genes, leading to alterations in ferroptosis pathways, which result in intricate impacts on malignant transformation and cancer progression in the liver [27, 35].

For example, LncRNA-D16366 was downregulated in HCC tissues and associated with tumor size and clinical prognosis [36]. LncRNA-D16366 could serve as a promising biomarker in HCC, which showed high diagnostic value, with an AUC of 0.752, a sensitivity of 65.5%, and a specificity of 84.6% [36]. NEAT1 formed a complex with U2AF65, which promoted cell proliferation, migration, and invasion in HCC models [37]. Overexpression of NEAT1 could enhance the anti-tumor responses by intensifying ferroptosis in HCC [38]. Additionally, the ferroptosis-related lncRNA NRAV impacted HCC progression through the miR-375-3P/SLC7A11 axis [39]. And dysregulated NRAV was associated with clinical prognosis, which could serve as a biomarker in HCC therapy [39]. Hence, thoroughly understanding the modulatory mechanisms of ferroptosis by lncRNAs in the realm of HCC could expedite the comprehension of HCC and promote drug discovery and development. 

In this review, we highlight the significance and prevalence of ferroptosis in HCC initiation and tumorigenesis. We specifically elaborate on how lncRNAs reshape ferroptosis pathway and emerging applications of targeting lncRNAs for HCC therapy, aiming to provide novel insights into novel personalized approaches for HCC treatment.

## Mechanisms of ferroptosis pathways in HCC

**Fig. 1:**
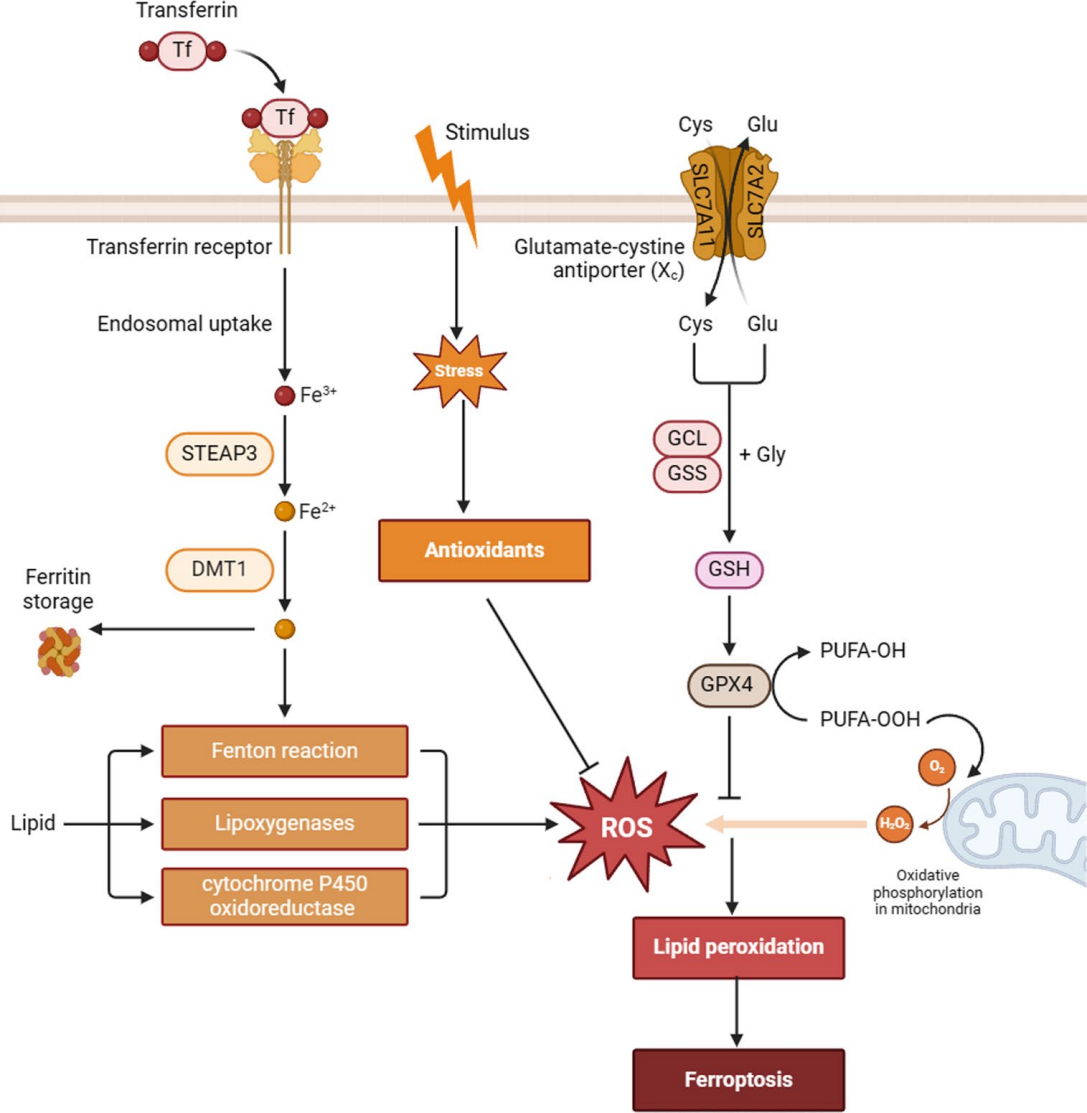
The signaling pathway of ferroptosis. The primary mechanism underlying ferroptosis is the initiation of iron-induced lipid peroxidation. System Xc- facilitates cystine uptake and glutathione (GSH) synthesis, while GPX4 is crucial for triggering lipid peroxidation. Extracellular iron enters cells mainly via the transferrin receptor (TFR), with intracellular free iron stored in ferritin, which involves the ferrpotosis pathway

Ferroptosis is driven by lethally accumulating phospholipid peroxides and intracellular iron overload in experimental models, involving a fragile equilibrium between cellular activities that promote ferroptosis and the antioxidant defense systems, generating toxic reactive oxygen species (ROS) and alkyl oxygen radicals, which in turn induce ruptured plasma membrane [40, 41] (Fig. 1).

The main aspect of ferroptosis is achieving the dynamic balance between oxidative and antioxidant damage [42]. Based on emerging evidence, the system Xc^−^- glutathione (GSH)- GPX4 pathway exerts significant effects on the modulation of ferroptosis [43]. The system Xc − , a cystine–glutamate antiporter which comprises subunits solute carrier family 3 member 2 (SLC3A2) and SLC7A11, are responsible for cystine import and GSH bioavailability [44, 45]. GSH functions as a reducing cofactor of GPX4 to reduce lipid peroxidation, which antagonizes ferroptosis in tumor cells [46]. Other antioxidant pathways, such as the endosomes as a requirement for transport III (ESCRT-III) membrane repair system, GTP cyclohydroxylase1 (GCH1)- tetrahydrobiopterin4 (BH4), as well as NAD(P)H-ferroptosis suppressor protein 1-ubiquinone (NAD(P)H-FSP1-CoQ10) all promote ferroptosis resistance in solid tumors [47, 48]. The canonical pathway triggers ferroptosis is suppressing the system Xc − via small molecules such as rat sarcoma viral oncogene homolog (RAS)-selective lethal 3 (RSL3), ferroptosis inducing 56 (FIN56) and erastin, resulting in the inhibition of GPX4 and causing GSH depletion, which further promote lipid peroxidation [49, 50].

More specifically, excessive iron accumulation is considered a precipitating factor of ferroptosis in experimental models [51, 52]. Intracellular iron overload is triggered by increased transferrin receptor-mediated cellular iron uptake and autophagic degradation of iron storage proteins (eg. ferritin) or iron export transporter SLC40A1 [51, 52]. Iron overload mediates lipid peroxidation by activating arachidonic acid lipoxygenases (ALOXs) [53]. During this process, phospholipids-polyunsaturated fatty acid (PL-PUFA) serves as the substrate, and following ROS production has driven the advanced oxidation process to mediate the production of phospholipid hydroperoxides (PL-PUFA-OOH) by ALOXs, which further promotes ferroptosis [54, 55].

Theoretically, cancer cells are characterized by robust metabolic action and massive ROS generation compared to normal cells, rendering them more vulnerable to ferroptosis [56]. Therefore, triggering ferroptosis holds great potential for cancer treatment, particularly for aggressive or treatment-resistant cancers.

## The molecular pathways of ferroptosis in HCC

### p62–Keap1–NRF2 pathway

NRF2, encoded by the NFE2L2 gene, is an essential transcription factor involved in oxidative stress, and mitochondrial function, as well as various pathophysiological processes [57, 58]. Under homeostatic conditions, NRF2 undergoes stringent regulation by Kelch-like ECH-associated protein 1 (Keap1) [59]. Keap1 functions as a substrate adaptor for cullin 3 (CUL3), which in turn recruits the E3-ubiquitin ligase RING-box protein 1 (RBX1) to target NRF2 and mediate its ubiquitination and degradation [59, 60]. However, in the presence of cellular stress, Keap1 undergoes conformational changes, blocking NRF2 degradation and allowing its nuclear translocation, leading to the transactivation of antioxidant genes, thus inhibiting ferroptosis [61, 62]. In HCC, NRF2 mutations accounted for approximately 15% of patients [63]. When HCC cells were exposed to ferroptosis inducers including sorafenib, buthionine sulfoximine, and erastin, there was an upregulation of p62 [64, 65]. Upregulated p62 exerts a regulatory crucial role by inactivating Keap1 and promoting the nuclear translocation of NRF2 [66]. Nuclear NRF2 transactivates HMOX1, NQO1, MT1G or ferritin heavy chain 1 (FTH1), which confers cellular defense mechanisms against ferroptosis [64, 65, 67]. Loss-function-analysis has shown that knockdown of p62 or NRF2-transactivated genes promoted ferroptosis upon exposure of HCC cells to erastin or sorafenib, while pharmacological suppression of NRF2 aggravated the antitumor effects of sorafenib [68, 69]. This process highlights the molecular mechanisms through which ferroptosis inducers impact the cellular regulation of p62–Keap1–NRF2 pathway in HCC cells [68, 69]. Therefore, the combination of sorafenib or erastin with genetic or pharmacological downregulation of p62-Keap1-NRF2 pathway might function as a promising approach to induce ferroptotic elimination of cancer cells.

### PI3K‐AKT-NRF2 pathway

Phosphoinositide 3-kinase/protein kinase B (PI3K/AKT) pathway plays a crucial role in regulating various cellular processes, including cell growth, survival, and metabolism [70, 71]. Mammalian target of rapamycin (mTOR) is a downstream effector of the PI3K/AKT cascade [72]. In the context of cancer, mTOR ensures the unlimited cell division, which is essential for cancer growth [72, 73]. mTOR forms two types of protein complex: mTORC1, and mTORC2, which are both implicated in cancer ferroptosis [74, 75]. mTORC2 engages in a direct interaction with SLC7A11, resulting in the phosphorylation of serine at position 26 on SLC7A11 [76, 77]. This phosphorylation event serves to inhibit the transporter activity of SLC7A11 [77]. Elevated cellular density triggers the activation of LATS1/2 kinase within the Hippo pathway, which in turn results in the phosphorylation of a constituent of mTORC1 [78]. This phosphorylation event inhibits activation of mTORC1, thereby blocking SLC7A11 degradation within the lysosome [78]. Furthermore, mTORC1 facilitates the interaction between p62 and Keap1, resulting in the nuclear accumulation of NRF2, which induces antioxidant defense proteins and glutathione synthesis enzymes including silence superoxide dismutase 1 (SOD1), GPX4, GSS and FTH1. This process suppresses the ferroptosis sensitivity to cancer cells [78, 79].

Sorafenib treatment activated PI3K-AKT-NRF2 axis was reported to upregulated ABCC5 expression in HCC cells [68, 80]. ABCC5 exerts impacts on drug resistance by expelling xenobiotics metabolites from cells [81, 82]. Enhanced ABCC5 facilitated SLC7A11 expression and decreased GPX4 depletion, which significantly suppressed ferroptosis by downgrading lipid peroxidation and increasing GSH levels [81]. Moreover, the upregulation of ABCC5 promoted the metabolization of sorafenib, thereby downregulating its intracellular toxicity [81]. Of note, pharmacological inhibition of ABCC5 facilitated ferroptotic elimination of HCC cells and reversed sorafenib resistance, which holds immense potential for HCC treatment.

### Hippo‐YAP pathway

Apart from the PI3K pathway which regulates cell growth and division, the Hippo pathway modulates organ size through the regulation of cell proliferation [83]. The Hippo pathway comprises a kinase cascade consisting of various protein kinases and transcription factors that are highly conserved [84]. E-cadherin (ECAD) serves as a crucial regulator of intercellular adhesion in epithelial cells, maintains cell polarity, and exhibits a positive correlation with cell density [85]. Disruption of the Hippo component or the overexpression of Yes-associated protein (YAP) impedes the reduction of cell proliferation induced by E-cadherin binding to the cell surface [86, 87]. The E-cadherin/catenin complex serves as an upstream regulator of the Hippo signaling pathway in mammalian cells and suppresses the activity of YAP within the nucleus, thereby directly modulating the Hippo pathway [87].

Emerging evidence has demonstrated that neighboring cells can activate the neurofibromatosis type 2 (NF2) and Hippo signaling pathways through ECAD-mediated interactions, thereby suppressing the ferroptosis of tumor cells [88]. The TEA domain transcription factor 4 (TEAD4), which interacts with Yes-associated protein (YAP) and enhances its activity, binds to the promoter regions of the ferroptosis markers TFRC and ACSL4. This effect is counteracted by the phosphorylation of YAP. Hence, an increase in cell density results in diminished expression of TFRC and ACSL4. This phenomenon can be reversed by the knockout of ECAD or NF2, or by inhibiting the phosphorylation of YAP, thereby promoting ferroptosis [89]. Furthermore, LATS1/2, a pivotal element of Hippo pathway, inhibits mTORC1 by phosphorylating Ser606 of Raptor, a key component of mTORC1, thus establishing the crosstalk between the Hippo signaling pathway and mTORC1 [90]. These findings suggest that the intercellular mechanism may serve as a critical cellular defense function against oxidative stress and ferroptosis.

## LncRNAs in HCC

LncRNAs exert a crucial impact on the complex regulation of gene expression [91]. The influence of lncRNAs extends across numerous levels of gene regulation, encompassing the restructuring of chromatin configurations and the facilitation of post-transcriptional modifications [92]. The core of their functionality lies their capability to engage with a diverse array of cellular components, including DNA, RNA, and proteins [93]. Through such interactions, lncRNAs emerge as vital regulators of various cellular processes [93]. Emerging evidence has revealed that lncRNAs play vital roles in multiple aspects of tumorigenesis [94, 95]. In the context of HCC, lncRNAs function as novel predictors of cancer recurrence and prognosis [96]. Moreover, lncRNAs significantly impact cell proliferation, migration, invasion and therapeutic response in the context of hepatocarcinogenesis [96]. As a critical lncRNA involved in the progression of HCC, metastasis-associated lung adenocarcinoma transcript 1 (MALAT1) is overexpressed in HCC tissues and regulates cell growth and metastasis [97, 98]. Moreover, MALAT1 is related with shorter overall survival of HCC patients [99]. More specifically, MALAT1 functions by binding and sequestering various miRNAs, thereby influencing cellular functions [100, 101]. For example, MALAT1 diminishes the expression of miR-204, leading to the upregulation of silent information regulator 1 (SIRT1) levels and the promotion of epithelial–mesenchymal transition (EMT) [102]. SIRT1 is a specific NAD + -dependent deacetylase that exerts significant impacts on the development of various diseases, making it a promising target for therapeutic intervention [103]. Additionally, MALAT1 enlists EZH2 to inhibit the expression of E-cadherin and miR-22, which significantly promotes EMT and tumor growth in HCC [104].

Angiogenesis, the formation of new blood vessels from pre-existing vessels, is a critical process in the rapid growth and hematogenous metastasis of HCC [105]. LncRNA PAARH promoted the hypoxia-inducible factor 1α (HIF-1α)/ Vascular endothelial growth factor (VEGF) pathway in HCC, facilitating angiogenesis and malignant progression [106]. Li et al. [107] found that lncRNA OR3A4 drove angiogenesis in HCC by modulating the AGGF1/AKT/mTOR pathway, which identifies OR3A4 as a promising therapeutic target for HCC. Moreover, lncRNA’s function also encompasses impacting drug resistance in HCC [108, 109]. NEAT1, an oncological lncRNA, has been demonstrated to contribute to sorafenib resistance, which is a key therapeutic agent for HCC [110]. In particular, NEAT1 inhibition by short hairpin RNAs (shRNAs) significantly enhances the effectiveness of sorafenib, leading to prompted drug-induced cell death and significantly smaller tumor mass in nude mice compared to treatment with sorafenib alone [111, 112]. Additionally, lncRNAs have the potential to serve as a supplementary treatment for immunotherapy, as they can augment immune cell responses while inhibiting tumor immune evasion [113, 114]. It was observed that lncRNA FENDRR inhibited miR-423-5p, thus impeding tumorigenicity, and Treg-mediated immune evasion in the context of HCC [115]. Fan and colleagues discovered a positive correlation between the levels of exosomal lncRNA PCED1B-AS1 in both blood and tissue and the expression of PD-L1 in HCC tissues [116]. Further mechanistic experiments have revealed that exosomal PCED1B-AS1 was released by HCC cells, which functioned as a molecular sponge for hsa-miR-194-5p [116]. Given that hsa-miR-194-5p negatively regulated PD-L1 expression, the inhibition of hsa-miR-194-5p by lncRNA PCED1B-AS1 led to the upregulation of PD-L1 expression, which might boost the immunotherapy efficacy in HCC [116].

AFP is a widely used biological biomarker for diagnosing HCC, playing a crucial role in early detection and disease monitoring [117, 118]. However, its diagnostic sensitivity is limited and its specificity is not optimal [117]. Therefore, it is essential to identify circulating biomarkers with high sensitivity and specificity. The importance of the lncRNA in the clinical diagnosis and prognosis of HCC has become apparent with advancements in recent studies [119, 120]. Dysregulated expression of several lncRNAs has been frequently observed in both HCC tissues and circulating fluids [120]. For instance, Chen et al. [121] used bioinformatics to construct robust lncRNA networks, highlighting the significant contribution of the lncRNA ZFAS1/hsa-miR-150-5p/GINS1 network to the early detection and disease monitoring of HCC. Furthermore, emerging evidence has identified that lncRNA DLX6-AS1 [122], MYCNOS [123], CRNDE [124], and LINC00221 [125] were closely associated with HCC patient prognosis.

In this context, we aim to provide a concise overview of how lncRNAs have the potential to remodel cellular signaling pathways, either inhibiting or exacerbating the progression of HCC. Gaining a deeper understanding of their involvement in various aspects of tumor progression and malignant development may contribute to better therapeutic responses in HCC.

## Ferroptosis-related lncRNAs in HCC progression and prognosis

Emerging research has revealed the involvement of ferroptosis-related lncRNAs in the initiation, progression, therapeutic response of HCC [126–128]. Certain tumor-suppressive lncRNAs promote ferroptosis to hinder HCC development and enhance the effectiveness of anticancer treatment, while oncogenic lncRNAs target molecules of ferroptosis pathway to promote the malignant progression of HCC (Table 1).

**Table 1:** Ferroptosis-related lncRNAs in HCC progression

LncRNA	Expression	Mechanism	Function	References
HULC	Up	HULC functioned as a ceRNA of miR-3200-5p and inhibited ferroptosis by targeting ATF4	Promoting cancer progression	[123]
lncFAL	Up	LncFAL decreased ferroptosis susceptibility through disrupting the FSP1-TRIM69 interaction and competitively inhibiting the polyubiquitination and degradation of FSP1	Promoting cancer progression	[126]
SNHG1	Up	SNHG1 increased FANCD2 and G6PD by sequestering miR-199a, leading to inhibited ferroptosis	Promoting cancer progression	[127]
DUXAP8	Up	DUXAP8 mitigated the susceptibility of HCC cell to sorafenib-mediated ferroptosis by reducing the membrane translocation of SLC7A11, promoting its lysosomal sorting and hindering its degradation	Promoting sorafenib resistance and cancer progression	[122]
GABPB1-AS1	Down	Erastin activated the transcription of GABPB1-AS1, reducing the expression of GABPB1, resulting in decreased PRDX5 expression and enhanced ferroptosis	Inhibiting cancer progression	[134]
HEPFAL	Down	HEPFAL enhanced the ubiquitination of SLC7A11 and reducing its stability by PI3K/AKT/mTOR pathway, leading to enhanced ferroptosis	Inhibiting cancer progression	[120]
NEAT1	Down	NEAT1 competitively sequestered miR-362-3p, resulting in a reduction of miR-362-3p-mediated MIOX inhibition, consequently intensifying ferroptosis	Inhibiting cancer progression	[135]
PVT1	Up	Ketamine triggered ferroptosis by regulating the lncRNA PVT1/miR-214-3p/GPX4 cascade	Inhibiting cancer progression	[137]

### LncRNAs inhibit ferroptosis to promote HCC progression

**Fig. 2:**
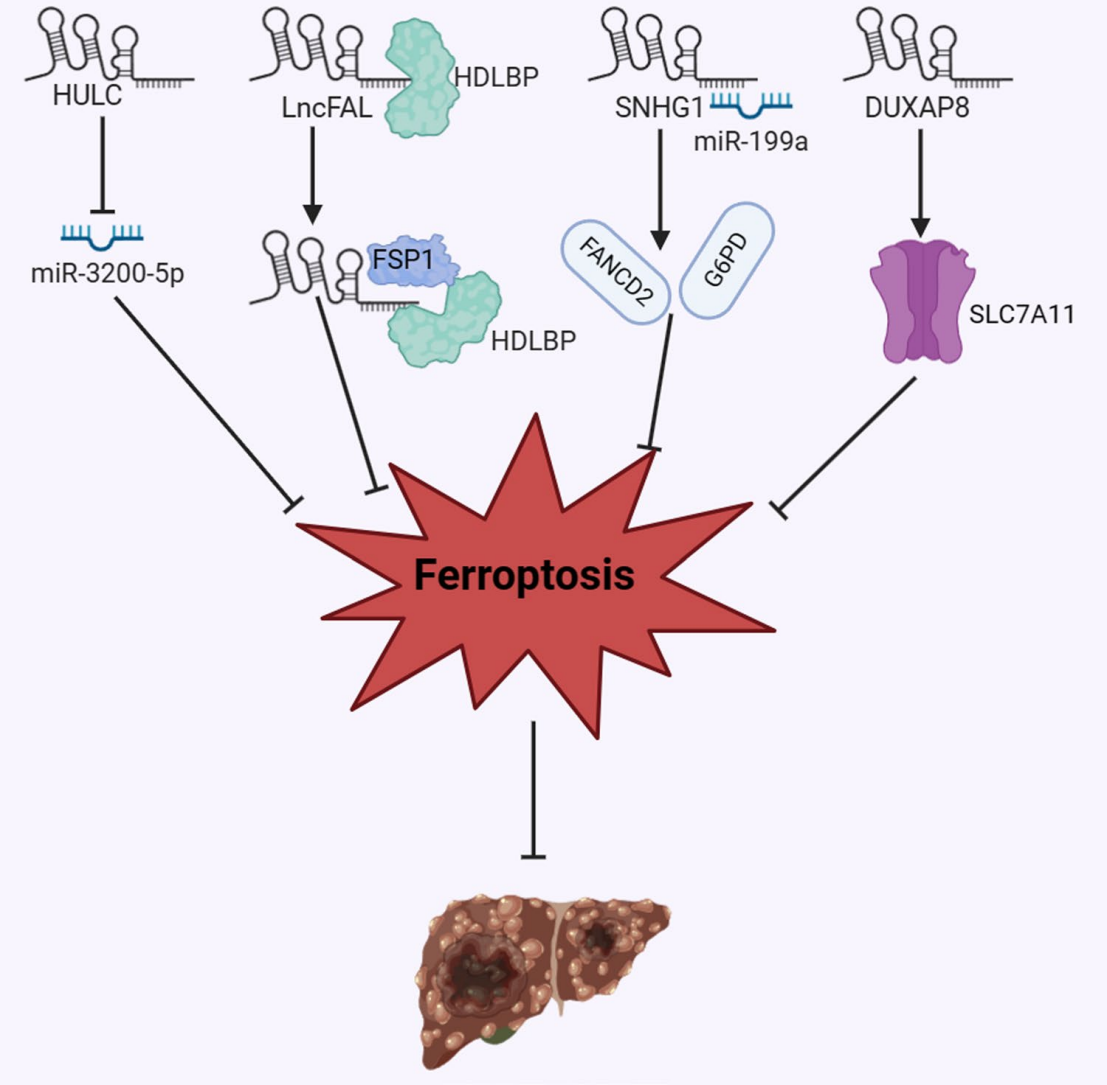
LncRNAs inhibit ferroptosis to promote HCC progression

Activating transcription factor 4 (ATF4), belonging to the ATF/cAMP response element-binding (CREB) family, serves as a crucial stress-induced transcription factor that negatively regulates ferroptosis [27]. Guan et al. [129] revealed that the depletion of lncRNA HULC elevated ferroptosis and oxidative stress in HCC cells. Additionally, HULC functioned as a competitive endogenous RNA (ceRNA) of miR-3200-5p and inhibited ferroptosis by targeting ATF4, leading to the promotion of cell proliferation and metastasis in HCC, suggesting the potential for targeting HULC/miR-3200-5p/ATF4 axis in HCC treatment [129] (Fig. 2).

High-density lipoprotein-binding protein (HDLBP) is a critical transporter that guards cells against excessive cholesterol accumulation [130]. Recent study has investigated its role in ferroptosis regulation. HDLBP was significantly upregulated in HCC tissues and this upregulation inhibited ferroptosis through stabilizing cytoplasmic ferroptosis-associated lncRNA (lncFAL) [131]. LncFAL decreased ferroptosis susceptibility through disrupting the ferroptosis suppressor protein 1 (FSP1)-TRIM69 interaction and competitively inhibiting the polyubiquitination and degradation of FSP1 [132]. These results advocate the considerable potential of targeting FSP1 as a promising therapeutic approach for HCC patients exhibiting upregulated expression of HDLBP or lncFAL, which also implies that targeting ferroptosis-suppressive lncRNAs could offer a novel therapeutic approach for HCC patients. Similarly, lncRNA SNHG1 increased Fanconi anemia complementation group D2 (FANCD2) and glucose-6-phosphate dehydrogenase (G6PD) by sequestering miR-199a, thus suppressing cell ferroptotic death in HCC [133].

Sorafenib is the first-line targeted drug for unresectable HCC, however, its clinical benefit has been significantly limited due to drug resistance [8]. Multiple studies have demonstrated that the anticancer efficacy of sorafenib is primarily attributed to its induction of ferroptosis and sorafenib resistance may stem from ferroptosis suppression [134]. LncRNA DUXAP8 exhibited high expression levels in HCC and correlated with poor prognosis of patients, which promoted sorafenib resistance by inhibiting ferroptosis [128, 135]. Mechanistic experiments demonstrated that DUXAP8 mitigated the susceptibility of HCC cell to sorafenib-mediated ferroptosis by reducing the membrane translocation of SLC7A11, promoting its lysosomal sorting and hindering its degradation in lysosomes, which actually activated the SLC7A11 action [128]. These processes suppressed sorafenib-induced ferroptosis and reduced sorafenib efficacy, while DUXAP8 knockdown inhibited tumorigenesis and reversed sorafenib resistance [128].

### LncRNAs promote ferroptosis to inhibit HCC progression

The GABPB1 protein serves as the activation subunit of the NRF2 transcription factor [136]. GABPB1 associates with the alpha subunit to form a tetrameric configuration that actively enhances the transcription of a myriad of genes, including those involved in antioxidant defense mechanisms, such as Peroxiredoxin 5 (PRDX5), which is highly expressed in HCC tissues and associates with poor prognosis of patients [137, 138]. The lncRNA GABPB1-AS1 is the antisense transcript of GABPB1 mRNA [139]. The expression of GABPB1-AS1 might fluctuate in response to stress stimuli [132, 140]. Qi et al. [140] have revealed that erastin activated the transcription of GABPB1-AS1, which significantly reduced the expression of GABPB1, resulting in decreased PRDX5 peroxidase expression. This interaction attenuated the antioxidant defenses of HCC cells and increased their susceptibility to ferroptosis and enhanced cell death [140]. Additionally, research by Zhang et al. [126] highlights the pivotal function of lncRNA HEPFAL in potentiating erastin-induced ferroptosis within HCC cells by enhancing the ubiquitination of SLC7A11 and reducing its stability. Further investigations revealed that this process was mediated by PI3K/AKT/mTOR pathway, resulting in inhibited tumor growth in nude mice, thereby presenting a potential new avenue for treating HCC [126]. LncRNA NEAT1 was identified as a direct target of p53, exhibited substantial upregulation in HCC cells undergoing ferroptosis induced by erastin or RSL3 [38]. NEAT1 competitively sequestered miR-362-3p, resulting in a reduction of miR-362-3p-mediated myo-inositol oxygenase (MIOX) inhibition, consequently intensifying the susceptibility of HCC cells to ferroptosis [38].

**Fig. 3:**
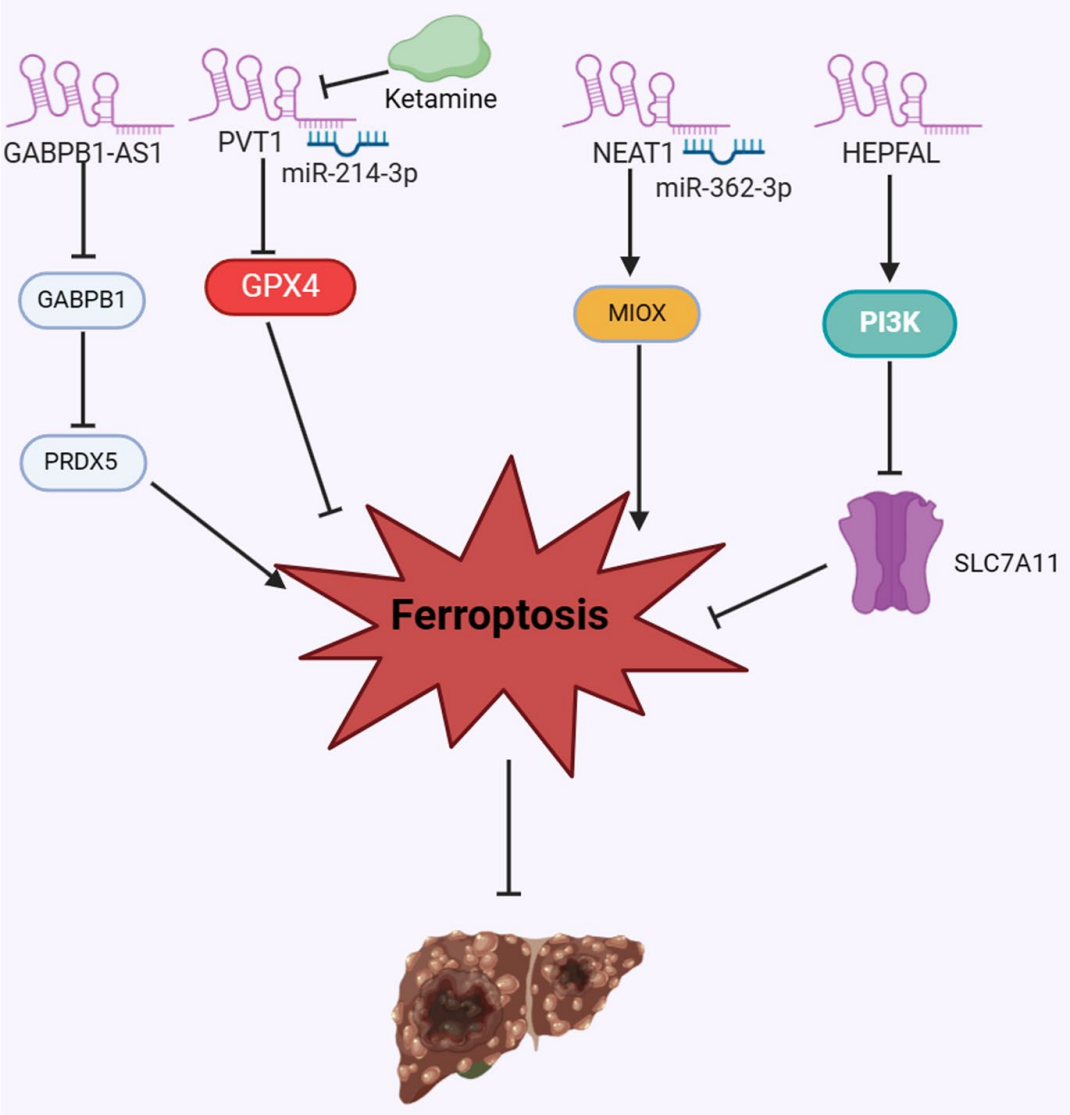
LncRNAs promote ferroptosis to inhibit HCC progression

Ketamine is a racemic blend comprising (S)- and (R)-ketamine that has been in clinically used since 1970 [141]. While widely recognized for its dissociative anesthetic qualities, ketamine also exerts analgesic, anti-inflammatory, and antidepressant effects [141]. In the context of HCC, Ketamine has been demonstrated to impede cell proliferation of HCC cells, facilitate apoptosis, and trigger ferroptosis by regulating the lncRNA PVT1/miR-214-3p/GPX4 cascade [142]. These findings revealed the potential significance of lncRNAs in fostering ferroptosis, suggesting a promising therapeutic strategy for HCC patients (Fig. 3).

### Ferroptosis-related lncRNAs as biomarkers in HCC.

LncRNAs exert an important role in the onset and advancement of HCC by modulating ferroptosis [38, 133]. It is evident that lncRNAs are involved in the activities of cell proliferation, invasion, EMT and tumor growth [30, 99]. Therefore, lncRNAs have potential prospects for clinical application as biomarkers for the diagnosis, treatment, recurrence, and prognosis of HCC.

LncRNAs could be detectable in the bloodstream of HCC patients [143]. Moreover, lcnRNAs expression are significantly dysregulated in the blood of HCC patients, suggesting a novel strategy for HCC diagnosis [143]. Studies have revealed that plasma level of HULC and NEAT1 are markedly elevated in HCC patients [144, 145], serving as significant predictors of HCC tumor growth and metastasis, which hold promise as non-invasive biomarkers for diagnosing hepatoma, and their diagnostic accuracy may be enhanced when combined with AFP [145–147]. Moreover, several studies have developed HCC prognostic models based on ferroptosis-related lncRNA signatures, aiming to enhance survival prognostication in HCC through tumor classification [127, 148]. These ferroptosis-related lncRNAs significantly contribute to the immunosuppressive tumor microenvironment and clinically therapeutic response in HCC, potentially aiding in individualized prognosis determination and treatment planning for HCC patients [127, 148]. For example, Wang and his colleagues have established a model based on ferroptosis-related lncRNAs that could accurately predict HCC prognosis, with associations to tumor grade, macrophage and fibroblast infiltration [149]. Mechanistic analysis revealed that these ferroptosis-related lncRNAs could exert significant impacts on the HCC immune microenvironment by modulating immune-related signaling pathways including the NF-κB and STAT5 pathways, ultimately influencing differentiation of immune infiltration [149]. Recently, Lin et al. [150] established a risk model based on 17 ferroptosis-associated lncRNAs for the individualized prognostic prediction of immunotherapy response in HCC patients. These findings suggest that ferroptosis-related lncRNAs may serve as promising markers for early detection, personalized treatment, and prognosis of HCC patients.

## Targeting strategies of lncRNAs in cancer therapy

Ferroptosis, a distinct form of non-apoptotic cell death, is implicated in various pathological conditions, including cancer [41]. Despite resistance to conventional therapeutic strategies, tumor cells are particularly vulnerable to enhanced cellular ferroptosis [19, 41]. Therefore, inducing ferroptosis presents a practical and potentially effective anticancer treatment strategy, especially for drug-tolerant cancers.

LncRNAs modulate ferroptosis-related pathways, leading to ferroptotic cell death [151]. LncRNA-based therapies have the potential for personalized cancer treatment due to tumor heterogeneity, and they may ultimately offer greater specificities than traditional ferroptosis-inducing agents. Emerging evidence has revealed that modulating lncRNA expression might be a promising avenue for cancer therapy [152, 153]. Emerging lncRNA-based therapeutic strategies include small interfering RNAs (siRNAs), antisense oligonucleotides (ASOs), and CRISPR/Cas9 technology [154, 155].

SiRNA is a type of RNA interference molecule consisting of 21–24 nucleotides, which functions by endonucleolytic cleavage of lncRNAs [156, 157]. SiRNAs directly induce the degradation of lncRNAs by recruiting the RNA-induced silencing complex (RISC) to interact with lncRNAs [157]. In lncRNA research, siRNAs have been effectively employed in various preclinical models to investigate the therapeutic potential of targeting specific lncRNAs [158, 159]. For instance, the siRNA-mediated knockdown of lncRNA CASC9 significantly decreased tumor formation in an HCC mouse model [160]. Meanwhile, siRNA targeting lncRNA NEAT1 notably suppressed cell proliferation, increased cell apoptosis, as well as arrested cell cycle [161]. Moreover, ASOs are short single-stranded DNAs that form a structural complex with specific lncRNAs, which can then be recognized and cleaved by RNase H [162]. Emerging evidence has indicated that ASOs and siRNA exhibit distinct silencing efficiencies, which are influenced by mutiple factors, including the subcellular location of lncRNAs [162, 163]. Lennox and colleagues have demonstrated that ASOs were more effective than siRNA for targeting nuclear lncRNAs, while siRNA was more efficient than ASOs in the cytoplasm [164]. This could be attributed to the predominant presence of RNase H in the nuclei, while RISC predominantly operates in the cytoplasm [165, 166]. LncRNA HLNC1 was upregulated in HCC samples and associated with cancer progression [167]. However, specifically ASO significantly reduced HLNC1 expression and effectively attenuated –tumor growth in the mouse model of HCC [167].

The CRISPR/Cas9 system comprises a single guide RNA (sgRNA) and a Cas9 enzyme [168]. The capacity of Cas9 to specifically bind and cleave DNA sequences makes it an immensely potent tool for genome engineering, and it has been extensively employed in diverse genomic studies over the past several years [168]. Due to its exceptional precision, effectiveness, enduring impact, and ease of customization, the CRISPR/Cas9 system has been effectively utilized for targeting lncRNAs, sparking increased research in the field of lncRNAs [168, 169]. CRISPR/Cas9 mediated silencing of lncRNA- RP11-156p1.3 significantly decreased cell viability and tumor growth in the context of HCC [170]. Nevertheless, CRISPR/Cas9 is unable to effectively silence all lncRNAs due to loci overlap [171]. In a genome-wide study, only 38% of lncRNAs were successfully silenced, while others had adverse effects on nearby genes [171, 172]. These commercial experiences in preclinical models using ASOs, siRNAs and CRISPR/Cas9 system provide a deep-set foundation for lncRNA-based therapeutics for HCC.

## Conclusions and future perspectives

In this review, we have emphasized the crucial role of lncRNAs in the progression of HCC by regulating ferroptosis. The present review has underscored the extensive and intricate nature of their interactions, underscoring the ongoing need for research in this emerging field. Sorafenib is a widely utilized chemotherapeutic agent in HCC, but drug resistance presents a significant challenge [108]. Attenuating HCC cell resistance to sorafenib may be achieved by employing ferroptosis inducers or inhibiting ferroptosis suppressors [8, 173]. Emerging lncRNAs have been demonstrated to impact malignant behaviors and sorafenib resistance in HCC through regulating ferroptosis [130, 173]. Moreover, ferroptosis-related lncRNAs could function as diagnosis and prognostic biomarkers for patients with HCC, although the identification of the most appropriate candidates for clinical prognosis poses a challenge. Therefore, gaining further insights into the interaction between ferroptosis and lncRNAs in the context of HCC, as well as elucidating the regulatory mechanisms of lncRNAs, hold immense potential to develop novel therapeutic strategies of HCC.

While the future appears promising for gene therapy that targets lncRNAs, there are persisting concerns about potential adverse effects, as it remains a developing concept and strategy in comparison to traditional medications. Due to the poor interspecies conservation of lncRNAs, many human lncRNAs are undetectable in mice [174]. Consequently, directly applying treatment techniques developed in vitro and animal models to human applications may be difficult, necessitating additional evaluation. Additionally, organ toxicity and off-target effects restrict the utilization of lncRNA-based therapeutic strategies for regulating ferroptosis in cancer [156]. Hence, it is essential to conduct ongoing research to address these challenges. Establishing a ferroptosis-associated lncRNA screening platform is necessary to identify lncRNAs specific to cancer and ferroptosis. In-depth investigation into the specific functions of lncRNAs in ferroptosis using advanced molecular biology techniques is also imperative. Notably, nanomedicine can be employed to mitigate the toxicity and adverse effects of lncRNA-based therapy [156, 170]. Moreover, an encouraging approach in this field involves utilizing artificial intelligence to expedite the high-throughput discovery and exploration of small molecules that interact with lncRNAs, which might pave the way for lncRNA-based therapeutics.

Interestingly, emerging evidence has revealed dual-targeted therapy based on lncRNA. LINC02936 interacted with sine oculis homeobox homolog 1 (SIX1) and recruited it to the promoter of ceruloplasmin, resulting in increased expression of ceruloplasmin, suppression of ferroptosis, and facilitation of cancer progression [175]. A small peptide administered to disrupt the LINC02936-SIX1 interaction effectively inhibited cancer progression by promoting ferroptosis [175]. Moreover, LINC00152 was identified as an oncogenic lncRNA in HCC by promoting cell proliferation, migration, and drug resistance [176, 177]. Saatci et al. [178] revealed that the unique combination of tamoxifen and inhibition of LINC00152 by shRNA prompted tamoxifen-dependent ferroptosis through the reduction of GPX4 and inducing Ca^2+^ overload via modulation of the PDE4D/cAMP/PKA/CREB pathway. This led to the generation of ROS, resulting in elevated Fe^2+^ levels and subsequent lipid peroxidation, which resulted in reversed tamoxifen resistance and remarkably impeded tumor growth in mice [178]. Subsequent investigations could explore potential alterations in sensitivity to ferroptosis-induced cell death in resistance to anticancer drugs, as well as the potential efficacy of targeting lncRNAs in overcoming resistance to these agents or in other resistant models susceptible to ferroptosis induction. These efforts can ultimately improve the prognosis of cancer patients.

## Data Availability

Data will be made available on request.
